# Different Clinical Presentations and Management in Complete Androgen Insensitivity Syndrome (CAIS)

**DOI:** 10.3390/ijerph16071268

**Published:** 2019-04-09

**Authors:** Lucia Lanciotti, Marta Cofini, Alberto Leonardi, Mirko Bertozzi, Laura Penta, Susanna Esposito

**Affiliations:** 1 Pediatric Clinic, Department of Surgical and Biomedical Sciences, Università degli Studi di Perugia, 06132 Perugia, Italy; lucia.lanciotti@gmail.com (L.L.); marta.cofini@gmail.com (M.C.); alberto.leonardi88@gmail.com (A.L.); laura.penta@ospedale.perugia.it (L.P.); 2 Pediatric Surgery, Azienda Ospedaliera Santa Maria della Misericordia, 20122 Perugia, Italy; mirkobertozzi@hotmail.com

**Keywords:** complete androgen insensitivity syndrome, disorders of sex development, hormonal replacement therapy, testicular germ cell tumour

## Abstract

Complete androgen insensitivity syndrome (CAIS) is an X-linked recessive genetic disorder resulting from maternally inherited or de novo mutations involving the androgen receptor gene, situated in the Xq11-q12 region. The diagnosis is based on the presence of female external genitalia in a 46, XY human individual, with normally developed but undescended testes and complete unresponsiveness of target tissues to androgens. Subsequently, pelvic ultrasound or magnetic resonance imaging (MRI) could be helpful in confirming the absence of Mullerian structures, revealing the presence of a blind-ending vagina and identifying testes. CAIS management still represents a unique challenge throughout childhood and adolescence, particularly regarding timing of gonadectomy, type of hormonal therapy, and psychological concerns. Indeed this condition is associated with an increased risk of testicular germ cell tumour (TGCT), although TGCT results less frequently than in other disorders of sex development (DSD). Furthermore, the majority of detected tumoral lesions are non-invasive and with a low probability of progression into aggressive forms. Therefore, histological, epidemiological, and prognostic features of testicular cancer in CAIS allow postponing of the gonadectomy until after pubertal age in order to guarantee the initial spontaneous pubertal development and avoid the necessity of hormonal replacement therapy (HRT) induction. However, HRT is necessary after gonadectomy in order to prevent symptoms of hypoestrogenism and to maintain secondary sexual features. This article presents differential clinical presentations and management in patients with CAIS to emphasize the continued importance of standardizing the clinical and surgical approach to this disorder.

## 1. Introduction

Androgen insensitivity syndrome (AIS) is an X-linked recessive genetic disorder that includes a group of metabolic syndromes with different degrees of androgen resistance [1,2]. It results from several mutations involving the androgen receptor (AR) gene situated in the Xq11-q12 region [1,2]. It is one of the most common causes of disorders of sex development (DSD) [3]. AIS could be divided into three different disorders depending on the degree of androgen insensitivity: complete AIS (CAIS), characterized by complete feminization of the external genitalia; partial AIS (PAIS), with a variable clinical presentation (mainly female, mainly male or ambiguous external genitalia); and mild AIS (MAIS), characterized by male external genitalia and impaired pubertal virilization [4,5].

CAIS is the most frequent manifestation of AIS and was first described by Morris in 1943 [6]. An AR gene mutation is found in more than 95% of patients with CAIS: 70% of them result from maternally inherited mutations, while the remaining 30% are de novo mutations [7]. It is characterized by feminization of the external genitalia in a 46, XY individual with unresponsiveness to androgen action and normally developed but undescended testes [8,9,10]. It is still considered a rare condition, with an estimated prevalence ranging from 1:20,400 to 1:99,100 male subjects [11]. PAIS results at least as common as CAIS; whereas the prevalence of MAIS has not yet been determined. However, it is much less frequently reported than CAIS and PAIS [12].

Genitalia virilization physiologically occurs between the 8th and 14th weeks of gestation and is strictly linked to androgen action and AR function [13]. Specifically, testosterone is responsible for the development of the epididymis, vas deferens and seminal vesicles from the Wolffian ducts, while other male genital structures, such as the prostate, penis, and scrotum, derive from the action of dihydrotestosterone [7,14]. On the other hand, during puberty, both adrenal and ovarian androgens favour the development of pubic and axillary hair in females, while adrenal and testicular androgens control the deepening of the voice, the enlargement of the penis and hair pattern development in males [7]. Additionally, the anti-Mullerian hormone (AMH) produced by the testes causes the regression of Mullerian ducts, preventing the formation of internal feminine genitalia [14]. Therefore, any type of alterations in the androgen pathway could lead to impaired virilization. This article presents differential clinical presentations and management in patients with CAIS to emphasize the continued importance of standardizing the clinical and surgical approach to this disorder.

## 2. Clinical Presentation

Patients with CAIS have normal female external genitalia with a 46, XY karyotype and undescended testes due to complete unresponsiveness towards androgen action. In fact, these individuals normally develop primordial testes in the abdomen during foetal life by the seventh week after conception due to the presence of the SRY region and start of testosterone production, whose action is not effective because of the AR mutation in target cells [7,9]. Therefore, these patients lack other male genitalia, except for testes. Additionally, internal female genitalia are also absent because the abdominal testes normally produce AMH, which impedes the development of the uterus, cervix and proximal vagina [9]. However, the distal part of the vagina can be observed because it is not under AMH control, but it is always shorter than normal and blind-ending [15,16]. In patients with CAIS, puberty typically appears later and has a slower advance than in the general female population. However, breasts and female adiposity can develop regularly due to the action of oestradiol deriving from the peripheral aromatization of testosterone [17]. In contrast, pubic and axillary hair is absent or very rare because it mostly depends on androgen action. In regard to final height, CAIS patients are typically taller than the healthy female population due to the presence of the Y chromosome, which intervenes on statural growth independently of hormonal status [4,18,19,20,21]. The typical hormone profile is characterized by a high level of luteinizing hormone (LH) above the usual reference range, while the follicle stimulating hormone (FSH) level is usually normal, probably due to gonadal inhibin regulation [22,23,24]. Moreover, the basal testosterone value results are typically within the normal male range but increased relative to the female range, while the oestradiol level is normal referring it to the male range but in the lower range for females [22,23].

Therefore, CAIS should be suspected in these cases, depending on the patient’s age: in a neonate with female external genitalia when a prenatal test showed a 46, XY karyotype; in a female child who presents with an inguinal hernia, which is very rare in girls, or with labial swelling containing testis; and, finally, at puberty, in females with primary amenorrhea [3,7,15].

Considering diagnostic imaging, pelvic ultrasounds or MRIs could be helpful in confirming the absence of Mullerian structures, revealing the presence of a blind-ending vagina and identifying testes. Finally, the diagnosis is based on clinical presentation, laboratory tests and imaging in a female with a 46, XY karyotype and confirmed throughout AR gene analysis.

The Ethics Committee of Umbria Region (CEAS) approved the publication of our cases (PED-2018-12). Written informed consent was obtained from the parents of the two enrolled patients and the two patients provided their written assent.

## 3. Androgen Receptor (AR) Gene and Protein

Androgens play a crucial role in both reproductive and non-reproductive male functions throughout the lifespan [25]. Indeed, during the foetal period, they are responsible for the correct development of internal and external genitalia while, during puberty, they modulate growth and functions of all components of the male genital system. They also allow pubertal spurts in males and the development of secondary sexual characters in both girls and boys. Finally, in adults, they regulate bone and muscle health, spermatogenesis, and fertility [26,27].

**Figure 1:**
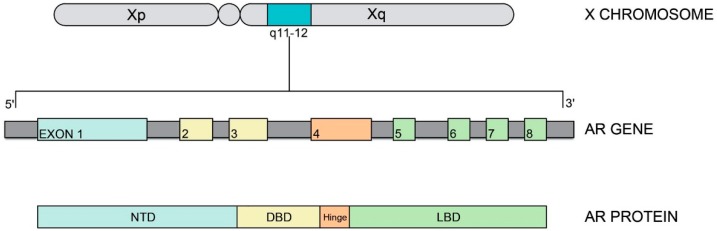
Androgen receptor gene and protein.

Androgen action mainly depends on the direct interaction with the AR [28], encoded by the AR gene, which is contained on the long arm of the X chromosome (Xq11-13). This gene is composed by 8 exons that encode a protein of 920 amino acid residues [29,30,31]. The AR is a member of the steroid hormone receptor family [28] and is a single-strand polypeptide consisting of four functional domains (Figure 1): (1) the N-terminal domain (NTD), (2) the DNA-binding domain (DBD), (3) the hinge domain, and (4) the C-terminal ligand–binding domain (LBD) [32,33]. The NTD region (~538 amino acids) is encoded by exon 1; it contains the action function-1 (AF-1) region and homopolymeric amino acid regions that hold polymeric repetitions of glutamine and glycine. Working together with other gene regions, these homopolymeric amino acid regions intervene in transcription regulation and define the three-dimensional final structure of the AR [17,29,34,35,36,37]. Furthermore, the length of the homopolymeric regions seems to be inversely related to AR transcriptional activity [38,39,40,41]. Large deletions of NTD have been associated with a significant reduction in transactivation capacity [42,43]. In particular, AF-1 plays a pivotal role in transactivation and regulates the interaction with LBD [44,45,46,47]. 

The DBD region (~558–617 amino acids) is encoded by exons 2 and 3, and it consists of two zinc-finger proteins: one binds to DNA, and the second modulates this interaction [5,48,49,50]. The overlapped region between the DBD region and the next hinge region enables the translocation of the activated AR from the cytosol to the nucleus [51].

Furthermore, the hinge region contains the phosphorylation site for AR and is responsible for the AR androgen-dependent structural changes. This region consists of ~618–637 amino acid residues and is encoded by part of exon [45,51,52,53]. The C-terminal LBD region (646–920 amino acids), encoded by exons 4–8, holds specific ligand-binding sites for the androgens, various transcriptional coactivator factors and the activation functional 2 (AF-2) region [51]. The AF-2 region is important for stabilizing the entire protein structure and allowing the interaction between the NTD region and specific coregulators [54].

Mutations in AR may lead to several abnormalities, such as a deficit/alteration in AR synthesis or an inability to bind the ligand [55]. Currently, approximately 900 different mutations have been associated with AIS according to an AR gene mutations database [12,56]. Four types of mutations have been widely investigated in patients with AIS: (1) single point mutations that result in stop codons or in amino acid substitutions, (2) deletions and insertions that lead to shifts in the translation reading frame, (3) partial or complete gene deletions involving a large part of the gene sequence, and (4) mutations that involve introns, altering RNA splicing [12,57,58].

Mutations involving the NTD region often lead to premature stop codons or to frameshift alterations [59], which have been frequently associated with CAIS [30]. Mutations detected in the DBD region instead lead to impaired activation of the AR through alterations in DNA-binding and/or dimerization activity [60]. However, two studies have suggested that some mutations involving this region do not lead to the loss of AR [61,62] functioning. Few mutations have been reported in the hinge region, perhaps due to its genetically determined flexibility/resistance or to the absence of gene sequences that have a significant impact on AR activity [63].

The largest percentage of mutations has been identified in the LBD region, which can impair several functions of the AR, such as AR stability, ligand and binding capacity and interaction with other coactivators. Mutations in this region have been associated with both CAIS and PAIS [63].

## 4. Time of Gonadectomy and Risk of Malignancy

In patients with CAIS the real need of gonadectomy is still debated: in fact on one hand the syndrome is associated with an increased risk of testicular germ cell tumour (TGCT), so gonads should be removed in order to prevent testicular cancer; on the other hand, the postponement of gonadectomy until at least puberty allows spontaneous pubertal development thanks to oestradiol deriving from the peripheral aromatization of testosterone produced by the retained testes.

TGCT represents approximately 1–1.5% of all tumours in the general male population and is the most common malignant cancer among male subjects from 15–40 years of age [64]. The occurrence of TGCT in adulthood could be above 22% [65], while its incidence is very low in childhood and adolescence [66].

According to the last published WHO classification, the majority of TGCT originates from noninvasive lesions, referred to as germ cell neoplasia in situ (GCNIS) and pre-GCNIS [67]. The CAIS condition has been related to a higher incidence of TGCT than in the general population [68]. The most common association is reported with seminoma and gonadoblastoma, although other histological forms have been found, such as choriocarcinomas, teratomas, embryonal tumours, adenomas, and Leydig and/or Sertoli cell tumours [69,70].

The exact incidence of cancer in patients with CAIS is very difficult to estimate because of the frequent change in management of this disorder over the years, particularly regarding the correct time of gonadectomy [71]. Data from the literature review report a general risk of approximately 5% in AIS disorder overall and a prevalence of <1% in CAIS [72,73]. In addition, the risk of malignant progression is elevated only with increased age [74,75]; indeed, it rarely occurs in prepubertal age (less than 1%), in contrast with other DSD, including PAIS [72,76]. In the general population, GCNIS advances into invasive cancer in approximately 50% of cases over five years [77], while the majority of malignant lesions described in patients with CAIS after puberty were pre-GCNIS or GCNIS, with a low likelihood of becoming invasive [68,71,78,79,80,81]. These data suggest that malignant progression from pre-GCNIS to invasive TCGT is very infrequent and probably takes place only in late adulthood. These findings validate the possibility of postponing a gonadectomy until after puberty [3,21,65,82,83,84,85,86]. Even the occurrence of a bilateral inguinal hernia during childhood no longer represents an absolute indication for early gonadectomy [75].

Several studies have tried to identify factors associated with cancer development and progression. It has been suggested, for example, that there is a possible role of individual genetic susceptibility, related to one or more single nucleotide polymorphisms (SNPs) [87,88]. Cools et al. (2017) found a significantly increased genetic susceptibility to the development of invasive cancer in subjects with pre-GCNIS due to the presence of specific alleles of genes related to invasive cancer. They did not find specific patterns of SNPs directly associated with pre-GCNIS/GCNIS/invasive cancer, but they stressed the possible role of genetic factors in cancer development together with residual androgen paracrine action and testicular cellular milieu [68]. Additionally, a higher risk of malignant transformation has been associated with altered expression of the histological markers PUO5F1 and KITLG [71]. POU5F1, also known as OCT3/4, represents a marker of delayed maturation of germ cells (early primordial germ cells), a condition commonly reported in a situation of insufficient hormonal action and/or defective cellular milieu, such as happens in DSD. Although an increased positivity to POU5F1 does not represent a premalignant condition ipso facto; the overexpression or the defective downregulation of POU5F1, particularly in germ cells in contact with the basal membrane, could promote the development of premalignant/malignant lesions by providing these cells with an increased survival capacity [73]. On the other hand, aberrant gene expression of KITLG has only been related to pre-GCNIS and not to the delayed maturation status of germ cells [89]. Therefore, the ability to distinguish the delayed maturation status of germ cells from premalignant lesions could allow for early identification of suspected lesions and overdiagnosis of GCNIS [80].

Furthermore, testis-specific protein, Y-linked (TSPY), could be another pivotal marker for malignant progression; indeed, it is physiologically involved in cellular proliferation [90,91,92,93]. Normal surviving germ cells in DSD usually overexpress TSPY, whereas its expression gradually decreases simultaneously with neoplastic progression until it becomes undetectable [94].

There may be several reasons for a low trend of malignancy in retained gonads in patients with CAIS. First, in contrast with other disorders of sexual development with gonadal dysgenesis, testicular tissue is normally developed in CAIS. Second, the lack of signal coming from androgens may play a key role in modulating cellular development and differentiation. Finally, the high rate of germ cell apoptosis in CAIS reduces the possibility of malignant evolution [68,71,73,95,96,97,98,99]. However, the residual paracrine actions of androgen in testicular tissue, also described in CAIS, could be a risk factor for cancer development, especially during and after puberty [97,98]. Indeed, it could promote neoplastic progression of germ cells and explain the increased risk of developing malignancy in adulthood [68,100]. On the other hand, some authors suggested the possible protective role of the residual androgen activity in cancer development, precisely because it allows the survival of the normal germ cell population overall [71,98,99].

Although there is a low rate of invasive cancer in CAIS, it is mandatory to recognize suspected lesions early. Unfortunately, both GCNIS and seminomas do not usually secrete serum markers, such as β-HCG and α-FP [101,102]; other specific serum markers are needed. For example, some microRNA clusters, such as the overexpression of miR371-3 and miR-302/367, have been associated with an invasive form of TGCT and with GCNIS both in DSD and in the general male population [103,104,105,106,107,108]. While these microRNAs have demonstrated promise both in the diagnosis and in the follow-up of TGCT, the GCNIS form likely does not secrete enough microRNAs to be useful for early diagnosis [104,109]. Currently, the real effect on the testes position still remains unclear as a promoting factor in cancer development in CAIS [71].

In summary, CAIS is a condition associated with an increased risk of cancer, although cancer results less frequently in CAIS compared to other DSD. The majority of tumoral lesions detected are non-invasive ones, with a low rate of progression into aggressive forms. Multiple factors seem to be involved, including individual genetic susceptibility, residual paracrine androgen effect, and testes position, and there are not any reliable serum markers to identify early lesions, though there are many suitable candidates. Nevertheless, histological, epidemiological, and prognostic features of testicular cancer in CAIS allow the postponing of gonadectomy until after pubertal age. 

## 5. Follow-Up of Retained Testes

Currently, about 15% of adult patients with CAIS decide to maintain their gonads intact, even after pubertal development [65]. This is probably due to the fact that they want both to take advantage of the benefits of endogenous hormone secretion and to avoid the possible complications of the surgical procedure [68]. Therefore, an effective follow-up programme is needed, in order to precociously recognize and afford the development of a TGCT. However, there is actually not a sufficient amount of confirmed data to guarantee safe management of these patients. 

Ultrasound (US) remains the first-line evaluation for inguinal or labioscrotal gonads [110], and annual US follow-up is recommended, starting from puberty [80]. US evaluation can identify suggestive lesions, such as microlithiasis and/or irregular echogenicity of testis parenchyma, but it is not able to properly detect GCNIS [111].

Magnetic resonance (MR) has to be performed in abdominal testes [110]. Although it is not able to identify GCNIS and/or microlithiasis [110,112], this procedure appears to be crucial for TGCT staging and follow-up. Nakhal et al. (2013) retrospectively evaluated testicular MR images of 25 patients with CAIS in order to investigate the effective role of MR in early identification of suspected lesions. MR was not predictive for the diagnosis of premalignant lesions, but it detected both paratesticular cysts and Sertoli cell adenomas [113]. Therefore, the possible role of MR in the identification of early invasive TGCT lesions remains debatable. Instead, Dohnert et al. (2017) proposed a biannual follow-up, including both US and/or MR, along with the determination of classic serum markers (e.g., β-HCG, α-FP, LDH) and hormonal assessment (FSH, LH, testosterone and Inhibin B) [75]. 

Further investigations are needed to detect how to perform the follow-up of patients with CAIS and unremoved testis after pubertal age. Currently, the gold standard for effective diagnosis of TGCT and/or its precursors still remains histological analysis at biopsy, which may result in gonadectomy [113,114].

## 6. Hormonal Replacement Therapy (HRT)

HRT is mandatory after bilateral gonadectomy in order to prevent symptoms of hypoestrogenism, inducing pubertal development if surgery has been performed before pubertal age or maintaining secondary sexual features if it has been performed later [115,116]. Secondary therapeutic targets of HRT also differ depending on the time of the gonadectomy; it allows physiological pubertal spurt development, physiological changes in body composition (fat and muscle mass distribution), achievement of bone mineral peak and maintenance of bone mineralization, and psychological and relational/sexual wellness [115,116].

The classic HRT for CAIS patients is based on oestrogen therapy, but current data are not able to indicate the best daily dosage. Therefore, HRT should be started at the lowest dose (i.e., oral ethinyl oestradiol 2.5–5 µg/day or 50–100 ng/kg/day) and then gradually increased to the adult dosage (i.e., oral ethinyl oestradiol 20–25 µg/day) in order to simulate physiological secretion [115,116,117]. Specifically in prepubertal subjects, HRT should be slowly increased every 6 months in order to complete feminization, such as breast development, changes in body composition and reaching of female body shape, in approximately two years [13,118]. After complete breast development, therapy should be continued with a regular daily dose [118,119]. As previously assessed, there is conflicting data about the optimal dose of oestrogen after the initial titration and, in particular, there are no specific trials conducted on CAIS subjects [115]. Furthermore, excessive doses could lead to impaired growth development and early epiphyses closure [115,120]. Therefore, HRT could be individualized according to clinical experience and patient needs. There is also no agreement on which is the best hormone formulation. Indeed, both oral and transdermal oestrogens seem to be useful and effective [115,116,117]; perhaps transdermal should be preferred to oral formulations for a more physiological delivery, an absent/lower first-pass effect, less interference with hepatic metabolism and IGF-1 serum levels, and a decreased risk of thromboembolism [121].

Furthermore, there are no consistent data on the real adverse effects of classic HRT in CAIS. Some studies have reported a slightly increased risk of myocardial infarction, stroke and breast cancer in adult menopausal women with oral administration [121], but the absolute risk seems to be very low, and these results could be useless for young patients with CAIS. However, in the literature, an increased risk of osteoporosis, cardiovascular diseases, dementia or cognitive decline, and Parkinson disease has been reported in subjects with early ovarian failure if untreated with oestrogen HRT [122].

The additional therapy with progesterone is not required because of the absence of a uterus in patients with CAIS, and there is no evidence of increased well-being with estroprogestinic therapy [15,115,116,117]. 

Despite the correct administration of classic HRT, many patients with CAIS reported a decrease in psychological well-being and in sexual satisfaction, perhaps due to several hormonal changes after the bilateral gonadectomy [123,124]. In a multicentre, double-blind and randomized crossover trial, the effectiveness and side effects of oestrogen versus testosterone HRT were investigated in 26 patients (ranging from 18–54 years old) genetically diagnosed with CAIS who had undergone a bilateral gonadectomy. No significant differences were found in terms of psychological well-being, mental health and quality of life between subjects who received oestrogen and those who received testosterone. Furthermore, no signs of virilization were observed with testosterone HRT, but it seemed to be better than oestradiol in improving sexual desire. Authors concluded that testosterone should be considered a valid alternative to oestrogen HRT in CAIS [123]. Future studies are needed to understand what could be the best therapeutic approach.

Interestingly, patients with CAIS seem to have a different hormonal status that does not follow a physiological male or female profile. In particular, postpubertal CAIS patients with intact gonads show increased levels of LH with normal levels of FSH and of sex hormone binding globulin (SHBG) for the female range; moreover, basal testosterone and oestradiol values, free androgen indices and androgen aromatization indices are in the normal male range [8,22,23,125]. Increased levels of LH, despite testosterone levels, may be attributable to the role of androgen resistance in the normal negative feedback action carried out by androgens on the hypothalamus-hypophysis axis [4,126]. Thus, Doenhert et al. (2015) suggested the use of a lower dose of HRT in patients with CAIS after gonadectomy, seeing that these patients follow neither a female nor male hormone pattern and that levels of oestrogen are normally below the female range before gonadectomy. This could partially explain the reported reduced wellbeing with doses of current classic HRT [23].

## 7. Bone Mineral Density and Body Composition

As previously assessed, androgens are involved in physiological body development, including achievement of bone mass peak and maintenance of bone mineral density (BMD) [127,128]. The presence of AR in osteoclasts, osteoblasts, osteocytes, and chondrocytes in the general male population confirms the pivotal role of androgens in bone homeostasis [128,129,130,131,132] Indeed, CAIS disorder seems to be associated with a reduced BMD on dual-energy X-ray absorptiometry (DXA) and an increased risk of osteoporosis in adulthood due to a lack of androgen function [78,133]; bilateral gonadectomy may also play an important role [134]. Certainly, the early identification of bone mineral density alterations could prevent comorbidity and improve the quality of life of these subjects. 

Decreased BMD in CAIS patients with removed gonads has been widely reported in the literature [21,135,136,137,138,139,140,141,142], while BMD seems to be less impaired in adult patients with intact gonads [21,133,140]. Moreover, lumbar BMD seems to be more affected than vertebral BMD, independent of gonadal status, suggesting a different pattern of AR expression between trabecular and compact bone tissue [129,132,143]. In patients who underwent gonadectomy, good adherence to HRT may play a role in BMD; indeed, it has been associated with better vertebral and femoral BMD levels at DXA [138,141]. In contrast, Danilovic et al. (2007) found, at most, a slight improvement in vertebral DXA values after two years of correct HRT administration [21], suggesting that other factors may be involved. Otherwise, a positive effect of HRT in BMD improvement could only occur after prolonged and/or high-dose therapy (i.e., equivalent to 0.625–1.25 mg of conjugated oestrogens) [142,144].

Regarding oestrogen formulations, transdermal administration could be more effective in achieving BMD improvement than an oral one, as is reported for Turner syndrome [145]. In a recent study conducted in patients with CAIS after gonadectomy, despite BMD impairment being detected both in vertebral and in hip DXA, it was unfortunately related neither to the time of gonadectomy nor to the type of therapy (oestrogen or testosterone), oestrogen formulation, or therapy adherence. Furthermore, no relationship between BMD and other hormones was found, including testosterone aromatization rate or oestrogen serum level. Finally, the normal range of BMD in patients with CAIS could differ from those currently used in the general female population because the higher mean height of patients with CAIS could play a role [146].

Some studies reported an increased fracture risk in patients with CAIS and removed gonads [136,138], but they involved only a small number of subjects, and often, there was substantial bias (i.e., reference values used for DXA), so the data are still inconclusive; there are no consistent data about fracture rate in patients with CAIS with intact gonads [134]. Additionally, patients with CAIS seem to also have a specific body composition; indeed, several animal studies have reported altered body fat mass with earlier development of obesity, an abnormal lipid profile, alterations in adipose tissue related hormones and decreased insulin sensitivity due to the resistance or absence of androgen activity [147,148,149,150]. Dati et al. (2009) have investigated body composition and metabolism assessment in middle-aged adult patients with CAIS, both with removed and retained testes. The body fat mass was increased and resulted in high values of total cholesterol and LDL cholesterol, and large amounts of HOMA-IR (Homeostatic Model Assessment for Insulin Resistance) were detected. Furthermore, they found an increased rate of obesity, even if the mean BMI did not differ significantly from the general female population of the same age. Interestingly, the majority of obese patients were those who retained testes. The authors suggested the importance of a regular assessment of body composition, metabolic status, and cardiovascular risk in all patients diagnosed with CAIS, regardless of gonadal condition [151]. Additionally, control of BMI and regular physical exercise are recommended together with calcium and vitamin D supplementation in order to improve bone health. Bisphosphonate therapy may be indicated only in the presence of a severely reduced BMD and/or fractures [17].

The specific effects of the increased levels of FSH on the osteoclasts, reported in CAIS patients with removed gonads [152,153,154], or the role of insulin-like factor 3 in gene-induced osteoblast differentiation, matrix apposition, and osteoclastogenesis could also be involved in BMD alterations [155] and should be thoroughly investigated.

## 8. Differential Diagnosis in Clinical Practice

**Figure 2:**
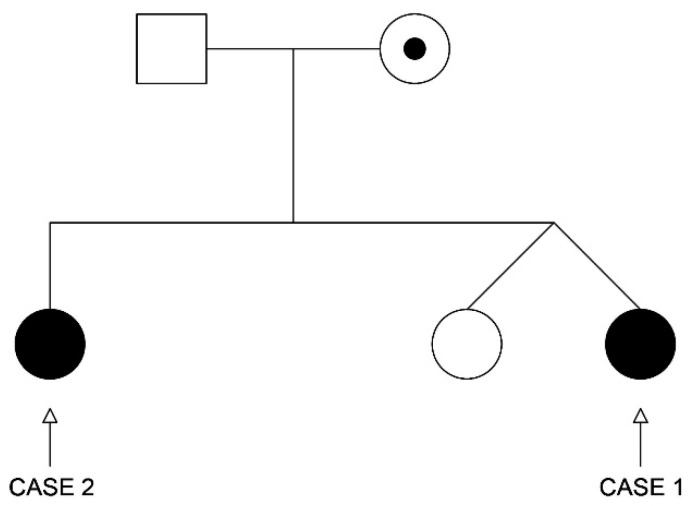
Genealogical tree of the family.

Below, we report the cases of two sisters with CAIS who underwent two very different methods of management (Figure 2). The Ethics Committee of Umbria Region (CEVAS) approved the publication of both cases. Written informed consent was obtained from the parents of the two enrolled patients and the two patients provided their written assent.

A. was referred to our Paediatric Endocrinology Unit at the age of 11.9 years old. Remote pathological anamnesis showed bilateral gonadectomy at one year of age for bilateral inguinal hernia. The surgical procedure showed the absence of both the uterus and ovaries together with a blind and hypoplastic vagina approximately 2.5 cm in length. Histological analysis demonstrated the presence of testicular tissue and the absence of abnormal cells or lesions. Therefore, karyotype analysis was performed and showed a complete male genotype, 46, XY. No other checks or investigations had been performed until our visit.

At our first evaluation, physical examination showed a female phenotype without any sign of pubertal development (Tanner stage 1 breast development). Her weight was 45 kg, and her height was 144 cm (−0.63 SDS), in agreement with mid-parental height (MPH), calculated with Tanner’s method for girls: (father’s height − 13 + mother’s height)/2 [156].

Regarding blood tests, serum LH, and FSH were elevated, while oestradiol and testosterone were undetectable. The other hormonal tests were in range (Table 1).

Considering diagnostic imaging, the bone age was congruent with her chronological age in a left-hand X-ray.

Genetic analysis of the AR gene was performed with MLPA and fragment analysis using a Genetic Analyzer and demonstrated hemizygous deletions involving both exon 4 and exon 5. No other genetic alterations were described. According to the Androgen Gene Receptor Database, the deletion of exon 5 is associated with CAIS [12], so the diagnosis of CAIS was confirmed. Therefore, psychological support was undertaken, and HRT with oestradiol hemihydrate patches was started at an initial low dose and then gradually increased. Currently, therapy is well tolerated with an absence of significant adverse effects, and A. is also continuing psychological support. After 10 months of therapy, she has stage 2–3 Tanner breast development.

Collecting a more accurate history, we found that A. had a twin sister and an older sister of 16 years with primary amenorrhea; we convened with the latter for evaluation (Figure 2).

I., the older sister of our first patient, came to our attention at the age of 16 years old for primary amenorrhea. Personal anamnesis showed breast development started at the age of 13 years, followed by growth of pubic and axillary hair. She had no relevant past medical history, and she denied medications. 

Her weight at the first evaluation was 53.5 kg, her height was 169.3 cm—1.23 SDS—and her BMI was 18.7 kg/m^2^. She was taller than her MPH of 150.35 ± 13 cm, as calculated by Tanner’s method for girls [156]. The physical examination showed neither major dysmorphia nor cutaneous or skeletal alterations. She had normal female external genitalia, her breasts were normally conformed (Tanner stage 3–4 breast development) and sparse pubic and axillary hair was present (Tanner stage 1 pubic hair). 

Regarding hormonal tests, serum LH (luteinizing hormone) and testosterone concentrations were elevated, while serum FSH (follicle stimulating hormone) and oestradiol concentrations were in the lower range of female values. The remaining hormonal tests were within the normal range (Table 2). The gonadotropin-releasing hormone (GnRH) stimulation test, which measures gonadotropin levels at different time points after GnRH administration, confirmed the suspicion of hypergonadotropic hypogonadism. 

Concerning diagnostic imaging, the bone age by X-ray agreed with the patient’s chronological age, while transabdominal ultrasound and subsequent pelvic MRI revealed the absence of anatomic structures compatible with the uterus and the presence of a blind-pouch vagina approximately 2.5 cm in length together with two oval homogenous structures within the pelvis.

The sex chromosome analysis demonstrated a complete male genotype, 46, XY, and SRY analysis showed the presence of the Y chromosome in all analysed cells. Then, the genetic analysis of the AR gene showed hemizygous deletions involving both exon 4 and exon 5, similar to her sister’s results. Therefore, the diagnosis of CAIS was confirmed and the first surgical evaluation was performed. Therefore, the patient underwent bilateral gonadectomy, with consent from her family. No alterations of the testes were found at histological analysis, and according to our patient, the gonads were also conserved.

After the excision of both testes, she started hormone replacement therapy with oestradiol hemihydrate patches at an initial low dose, which gradually increased. This therapy is well tolerated, with an absence of significant adverse effects, and I. is continuing her surgical follow-up in order to undergo vaginal dilatation and has even undertaken psychological support. 

Additionally, the genetic analysis was extended to the other female members of the family. The mother’s AR gene analysis showed heterozygote deletions of exon 4 and exon 5—the same as her daughters—while the AR gene of the twin sister of A. was normal, and she had a normal 46, XX female karyotype (Figure 2).

Unfortunately, we could not extend this analysis to the other members of the family because they still lived in their home country. 

## 9. Challenges in Diagnosis and Management

The diagnosis of CAIS still represents a demanding challenge and is often delayed until the evidence of primary amenorrhea during puberty, except for the cases in which bilateral inguinal hernia appears during childhood [3]. In this paper, we report the cases of two sisters diagnosed with CAIS following a very different presentation. In the first case A. had undergone bilateral gonadectomy and karyotype analysis at one year of age, but no other tests, such as AR gene analysis, were performed. In addition, genetic counselling was not considered for the other members of her family, so her sister I. was not diagnosed until 16 years of age.

Currently, all data from the literature agree with postponing gonadectomy until at least puberty in order to allow spontaneous pubertal development and avoid induction of puberty [75]. Therefore, gonadectomy should be avoided even in patients presenting evidence of an inguinal hernia in the first years of life in phenotypic female children, as in our first case. Nevertheless, genetic analysis involving a karyotype and the AR gene is mandatory in order to confirm the diagnosis and establish proper endocrinological, surgical, and psychological management. For example, our second patient, who underwent a gonadectomy in adolescence, only needed hormonal replacement therapy, considering her spontaneous pubertal development.

In our cases, genetic analysis showed deletions involving both exon 4 and exon 5 of the AR gene inherited from their mother, as in 70% of the cases described in scientific articles [7]. In the literature, the largest percentage of AR gene mutations involve the LBD region, to which exon 4 and 5 belong. In fact, a mutation in this region is particularly important because it can impair several AR functions, such as stability and ligand binding capacity [63].

According to current knowledge, exon 4 deletions could be associated both with CAIS and PAIS phenotypes, while exon 5 deletions are more frequently associated with CAIS, as reported in the AR gene mutation database. Specifically regarding exon 4, this database reports 6 different deletions and one deletion/insertion associated with CAIS and 2 different deletions associated with PAIS [11,12,100,157,158,159,160,161,162]. On the other hand, considering exon 5, there are nine different deletions and two deletion/insertions associated with CAIS [78,100,162,163,164,165,166,167,168,169,170] and none associated with PAIS.

## 10. Conclusions

In this manuscript we showed that CAIS management still represents a unique challenge throughout childhood and adolescence, particularly regarding timing of gonadectomy, type of hormonal therapy, and psychological concerns. Additionally, we demonstrated that genetic analysis should not be delayed because early diagnosis is important at any stage of life in order to establish proper endocrinological and surgical management. Moreover, we emphasized the importance of expanding genetic analysis to all female members of the family as well.

## Figures and Tables

**Table 1:** Serum hormone concentrations in Patient 1 at diagnosis.

Hormone	At first Evaluation	Reference Values
LH (mUI/mL)	23.82	2.3–3.5
FSH (mUI/mL)	130	2.4–5.2
Testosterone (ng/mL)	0.16	0.10–0.75
Oestradiol (pg/mL)	<20	21–85
Prolactin (ng/mL)	26.9	3.3–26.7
DHEA-S (ug/dL)	203.6	15–260
4-Androstenedione (ng/mL)	0.7	0.24–0.38
TSH (mUI/mL)	1.76	0.34–5.6
FT4 (ng/dL)	0.77	0.54–1.24

LH: luteinizing hormone, FSH: follicle stimulating hormone.

**Table 2:** Serum hormone concentrations in Patient 2 at diagnosis and after surgery.

Hormone	At Diagnosis	After Surgery	Reference Values
LH (mUI/Ml)	25.81	34.57	5.3–10.5
FSH (mUI/mL)	3.8	105.20	5.8–8.6
Testosterone (ng/mL)	4.9		0.10–0.75
Oestradiol (pg/mL)	23	48	21–85
Prolactin (ng/mL)	15.5		3–24
17-Hydroxyprogesterone (ng/mL)	1.5		0.16–2.31
DHEA-S (µg/dL)	261.8		35–535
4-Androstenedione (ng/mL)	4		0.3–3.5
β-HCG (mUI/mL)	1.92		0–5
α-Fetoprotein (ng/mL)	1.4		0.6–8.1
TSH (mUI/mL)		1.540	0.34–5.6
FT4 (ng/dL)		0.72	0.54–1.24
